# Detection of Microbial 16S rRNA Gene in the Serum of Patients With Gastric Cancer

**DOI:** 10.3389/fonc.2019.00608

**Published:** 2019-07-09

**Authors:** Zhaogang Dong, Bin Chen, Hongwei Pan, Ding Wang, Min Liu, Yongmei Yang, Mingjin Zou, Junjie Yang, Ke Xiao, Rui Zhao, Xin Zheng, Lei Zhang, Yi Zhang

**Affiliations:** ^1^Department of Clinical Laboratory, Qilu Hospital of Shandong University, Jinan, China; ^2^Shandong Province Key Laboratories of Medicine and Health (Tumor Marker Translational Medicine Laboratory), Qilu Hospital of Shandong University, Jinan, China; ^3^Beijing Advanced Innovation Center for Big Data-Based Precision Medicine, Beihang University, Beijing, China; ^4^College of Life Science, Qilu Normal University, Jinan, China; ^5^Shandong Children's Microbiome Center, Qilu Children's Hospital of Shandong University, Jinan, China; ^6^Qingdao Human Microbiome Center, The Affiliated Central Hospital of Qingdao University, Qingdao, China

**Keywords:** gastric cancer, microbiome, 16s rRNA gene, dysbiosis, serum

## Abstract

Aberrance in the blood bacterial microbiome has been identified and validated in several non-infectious diseases, including cancer. The occurrence and progression of gastric cancer has been found to be associated with alterations in the microbiome composition. However, the composition of the blood microbiome in patients with gastric cancer is not well-characterized. To test this hypothesis, we conducted a case-control study to investigate the microbiota compositions in the serum of patients with gastric cancer. The serum microbiome was investigated in patients with gastric cancer, atypical hyperplasia, chronic gastritis, and in healthy controls using 16S rRNA gene sequencing targeting the V1-V2 region. Our results revealed that the structure of the serum microbiome in gastric cancer was significantly different from all other groups, and alpha diversity decreased from the healthy control to patients with gastric cancer. The serum microbiome correlated significantly with tumor-node-metastasis (TNM) stage, lymphatic metastasis, tumor diameter, and invasion depth in gastric cancer. Three genera or species, namely, *Acinetobacter, Bacteroides, Haemophilus parainfluenzae*, were enriched in patients with gastric cancer, whereas *Sphingomonas, Comamonas*, and *Pseudomonas stutzeri* were enriched in the healthy control. Furthermore, the structure of serum microbiota differed between gastric cancer lymphatic metastasis and non-lymphatic metastasis. As a pilot investigation to characterizing the serum microbiome in gastric cancer, our study provided a foundation for improving our understanding of the role of microbiota in the pathogenesis of gastric cancer.

## Introduction

Gastric cancer (GC) is the third leading cause of cancer-related deaths worldwide, accounting for more than 720,000 deaths every year (1), therefore, elucidation of its etiology and pathogenesis is critical for reducing the mortality rate and improving the prognosis of patients with GC. However, the etiological factors and pathogenetic mechanisms involved in GC progression are complicated, multifactorial, and multistep (2). Host genetics, environmental influences, and dietary patterns are recognized as the most significant contributory agents for GC. In addition, the gut microbiota is also frequently suspected as another critical factor participating in the occurrence and progression of GC.

The human microbiota is an important microecosystem living symbiotically in the body (3). It plays an important role in multiple biological functions, and shifts in the composition of microbiota have been shown to contribute to the occurrence and development of GC. Considering the structural complexity and vital functions of the microbiota, the correlation between microbiota and GC has been investigated. *Helicobacter pylori* infection, identified by Marshall and Warren (4), plays a crucial role in the development of GC (5). However, only 3% of infected individuals develop GC (6). In addition, the successful eradication of *H. pylori* does not completely prevent GC development (7). These observations suggest that factors other than *H. pylori* contribute to GC progression. Recent advances in molecular techniques and bioinformatics have uncovered a complex microbiota within the stomach, which possesses the potential for disease induction (8). Studies have demonstrated that the diversity of the microbiota is reduced in patients with GC, and the combination of certain taxa can be used to differentiate between gastritis and gastric carcinoma (9–11). The diversity and composition of gastric microbiota is identified as a crucial determinant of inflammation and as a key player in GC. However, until now, information regarding the characteristics of the blood microbiome of patients with GC is scarce.

Blood is considered a “sterile” environment, and does not show bacterial growth in non-communicable diseases. However, with advancements in molecular techniques and bioinformatics, for example, 16S ribosomal RNA gene sequence, the blood bacterial microbiome has recently been identified and validated in non-infectious disease states. Potgieter et al. (12) considers that blood “non-culturable” microbes originate from the gut microbiome or oral cavities, and are dormant in blood. Aberrations in blood microbiota have been implicated in several non-infectious diseases. For example, Qian et al. revealed that *Isoptericola, Cloacibacterium, Enhydrobacter*, and *Microbacterium* were enriched in the blood of patients with Parkinson's disease, and were positively associated with disease duration. Fifteen blood genera were selected as markers, achieving good diagnostic value, with an area under the curve (AUC) of 0.686 and specificity of 95.6% (13). Olde Loohuis et al. also observed an increase in blood microbial diversity in patients with schizophrenia (14). Another study showed that the presence of Proteobacteria in blood was directly associated with the onset of cardiovascular disease (15). Therefore, blood microbiota might play an important role in disease development and progression. Recently, serum microbiome has attracted the attention of researchers. Dinakaran et al. (16) detected circulating cell-free DNA in patients with cardiovascular diseases using metagenomic shotgun sequencing, and observed a dominance of Actinobacteria, followed by Proteobacteria. Subsequently, Santiago et al. (17) observed that Firmicutes (41%), Bacteroidetes (37%), and Proteobacteria (14%) were present in the serum of patients with cirrhosis, and the serum microbiome was considered a live indicator of the progression of cirrhosis. Thus, the release of bacterial DNA elements in circulation may be involved in disease progression. However, to our knowledge, no study has yet investigated the serum microbiome in patients with GC.

Hence in this study, 311 serum samples from Qilu Hospital of Shandong University were collected. Finally, 215 samples were selected and subjected to 16S rRNA gene amplicon sequencing. The serum microbiome of healthy controls, and patients with atypical hyperplasia (AH), chronic gastritis (CG), and GC were successfully characterized. We also analyzed the relationships between the serum microbiome and clinical characteristics of GC. The aim of the present study was to characterize the microbial community in gastric cancer and investigate its potential associations with carcinogenesis.

## Materials and Methods

### Participant Information

In total, 311 serum samples from Qilu Hospital of Shandong University were collected from December 2016 to December 2017. GC was diagnosed through the integration of imaging, clinical symptoms and physical signs, laboratory tests, and medical history. GC was confirmed using the gold standard method of histopathological examination of specimens from surgical resection. Disease stages were defined according to the Union for International Cancer Control (UICC)/American Joint Committee on Cancer (AJCC) tumor-node-metastasis (TNM) staging system (2010). The exclusion criteria were: (1) the presence of a gastric stromal tumor, (2) prior treatment with proton pump inhibitors (PPI) or antibiotics within 1 year, (3) presence of other diseases affecting the immune system or hepatitis, (4) history of other malignancy, (5) missing clinical information of the participant, (6) presence of active infection when joining the study, (7) symptoms at physical examination or analytical data suggesting infection, and (8) an unwillingness to participate in this study.

Fifty healthy volunteers who visited our hospital for their annual physical examination were enrolled as controls. The inclusion criteria were: (1) normal physical examination, (2) normal results for routine examination of blood, urine and stool, erythrocyte sedimentation rate, liver function, renal function, electrolyte, blood glucose, and lipids, and (3) normal results of imaging examination (liver ultrasound and chest X-ray) and electrocardiogram. The exclusion criteria included the presence of hypertension, diabetes, obesity, irritable bowel syndrome (IBD), gastric or coeliac disease, and individuals who received antibiotics and/or probiotics within 1 year prior to enrolment. Our study was approved by the Ethics Committee of Qilu Hospital of Shandong University (KYLL-2015-097). The participants provided written informed consent in accordance with the Declaration of Helsinki.

### Samples Processing

Five milliliters of venous blood was drawn from each individual in 5-mL vacutainer tubes (SST^TM^ II, BD-Belliver industrial Estate, Plymouth, UK) on an empty stomach in the morning, and hemolysed blood samples were excluded from the study. To avoid potential contamination, we used gloves and followed aseptic procedures. For complete clotting, the tubes were incubated at room temperature (20–25°C) for 30 min to 1 h. After centrifugation at 3,000 × *g* for 10 min, serum was transferred into 1.5 mL sterile eppendorf tubes and stored as separate aliquots at −80°C for future use.

### DNA Extraction and Polymerase Chain Reaction (PCR)

To identify possible contamination in low-biomass samples, we introduced negative controls (blanks) in DNA extraction. QIAamp^®^ UCP pathogen mini kit (Cat no: 50214) was used to extract and purify DNA from 1.5 mL serum according to the manufacturer's instructions with minor modifications. In brief, the serum sample was centrifuged at 20,000 × *g* for 10 min. The supernatant was removed, and the pellet was suspended in 400 μL buffer ATL. Next, 40 μL proteinase K was added and the mixture was incubated at 56°C for 10 min. Subsequently, 200 μL buffer APL2 was added to the sample and incubated at 70°C for 10 min. QIAamp UCP mini spin column was used to purify DNA. After washing the column with 600 μL buffer APW1 and 750 μL buffer APW2. The assembly was incubated at 56°C for 3 min with open lid. Twenty microliter buffer AVE was applied to QIAamp UCP Mini membrane, and was centrifuged at 20,000 × *g* for 5 min for eluting DNA. DNA concentration was detected at 260 nm by NanoDrop ND-2000 (Thermo Fisher Scientific).

Two universal bacterial 16S rRNA gene amplicon PCR primers were used (V1-V2 region): forward primer-27F (5′-AGAGTTTGATCMTGGCTCAG-3′) and reverse primer-355R (5′-GCTGCCTCCCGTAGGAGT-3′). The PCR products were checked using electrophoresis in 1% (w/v) agarose gels in TBE buffer (Tris, boric acid, EDTA) stained with Genecolour I^TM^ (Gene-bio) and visualized under UV light. PCR products were pooled and purified using VAHTS^TM^ DNA clean beads (Vazyme Biotech) according to the manufacturer's instructions. All amplicons were quantified, and equal amounts of each sample were pooled for sequencing using HiSeq 2500 (Illumina). UV was used to clean consumables and H_2_O to remove potential sources of contamination. Negative controls (blanks) were also used.

### 16S rRNA Gene Amplification Sequence Processing

The FLASH method described by Magoč and Salzberg was used for splicing 16S rRNA gene sequence paired-end data set and for quality filtering (18). All sequences were analyzed using the Quantitative Insights Into Microbial Ecology (QIIME, version 1.9.1) software suite (19), based on the QIIME tutorial (http://qiime.org/) with some modifications. Usearch61 (20) with the *de novo* model was used to remove chimeric sequences. Sequences were clustered against the 2013 Greengenes (13_5 release) ribosomal database's 97% reference data set. UCLUST was used to subsequently cluster sequences that did not match any entries in this reference into *de novo* operational taxonomic units (OTUs) at 97% similarity. The RDP classifier (21) within QIIME and the Greengenes reference data set was used to assign taxonomy to all OTUs. The OTU tables were subsequently filtered based on abundance, removing low-abundance OTUs representing <0.005% of the total reads. Then, the OTU table was rarefied to a sequencing depth of 24,000 per sample for subsequent analyses of alpha and beta diversity. The QIIME standard pipeline was used to analyze alpha diversity and beta diversity.

### Fecal Occult Blood Tests

The colloidal gold-based fecal occult blood diagnostic kit (Chemtron Biotech Co. Shanghai, China) was used preoperatively according to the manufacturer's instructions to detect gastrointestinal bleeding in all cases.

### Bioinformatics and Statistic Analysis

The distribution of samples in each group was determined by the Kolmogorov-Smirnov test. The data was characterized using median values and ranges. The Kolmogorov–Smirnov test or Kruskal–Wallis test was used to evaluate the difference, as appropriate. QIIME was used for analyzing similarities (Adonis) on beta diversity matrices, to determine significant differences among microbial communities. The significance of the Adonis test was assessed with 9,999 permutations. Linear discriminant analysis effect size (LEfSe) was introduced to identify bacterial biomarkers for the two groups, performed on the Galaxy web-based interface (http://huttenhower.sph.harvard.edu/galaxy). We used standard parameters, with the exception of the alpha value for the factorial Kruskal–Wallis test among classes (alpha <0.05). Spearman's rank correlation was used to determine the statistical dependence between continuous variables. The false discovery rate (FDR) correction was used for multiple tests. Subjects with or without GC were distinguished using receiver operating characteristic (ROC) analysis. AUC was also calculated. The optimal cutoff value was determined by the Youden index (sensitivity+specificity−1). Statistical analyses were performed using SPSS V.25.0 for Windows (Chicago, Illinois, USA) and Medcalc software (Version 8.0, Korea). *P* < 0.05 was considered statistically significant.

## Results

### Characteristics of the Studied Cohort

**Figure 1:**
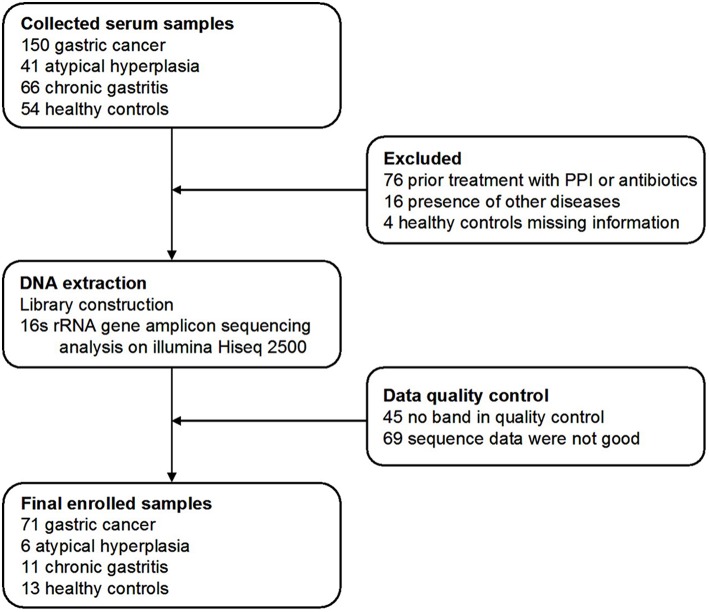
Study design and flow diagram. In total, 311 serum samples from Qilu hospital of Shandong University were collected. After a strict pathological diagnosis and exclusion process, the remaining samples were used for 16S rRNA gene sequence and data quality control. One hundred and one samples were finally used for bioinformatics analysis, including 71 gastric cancer, 6 atypical hyperplasia, 11 chronic gastritis samples, and 13 healthy controls.

Three hundred and eleven cases were enrolled in this study. After a strict pathological diagnosis and exclusion process, 215 serum samples were subjected to 16S rRNA gene sequencing. The samples included 50 healthy controls (HC) (25 males, age ranges 29–58 years. 25 females, age ranges 30–69 years), 34 chronic gastritis (CG) (17 males, age ranges 40–69 years. 17 females, age ranges 33–88 years), 23 atypical hyperplasia (AH) (18 males, age ranges 33–76 years. 5 females, age ranges 41–74 years), and 108 GC (83 males, age ranges 29–80 years. 25 females, age ranges 32–73 years). After data quality control, which excluded cases with no amplification of product or low-quality sequence data, the serum microbiome of 101 cases were finally subjected to bioinformatic analysis, including 71 GC, 6 AH, 11 CG, and 13 HC cases (Figure 1). Patients with GC were staged including stage 0, stage I, stage II, stage III, and stage IV. The characteristics of the patients with GC are summarized in Supplemental Table 1.

### Serum Microbiome in Patients With CG, AH, GC, and HC

**Figure 2:**
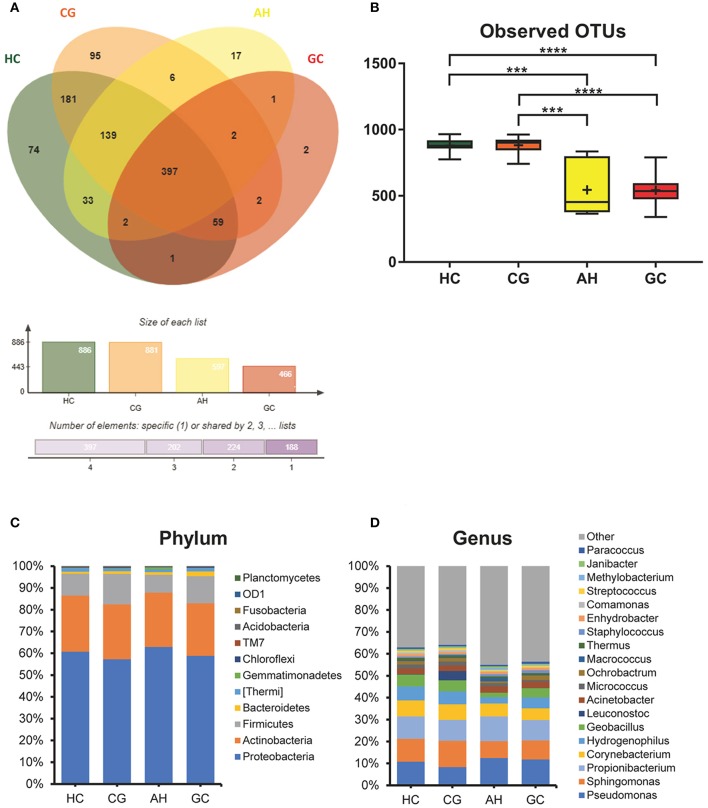
Comparison of serum microbiome among healthy controls (HC), and patients with chronic gastritis (CG), atypical hyperplasia (AH), and gastric cancer (GC). **(A)** Venn figure. **(B)** Observed OTUs among HC, CG, AH, and GC. **(C)** Barplots of the taxonomic profiles among HC, CG, AH, and GC at the phylum level. **(D)** Barplots of the taxonomic profiles among HC, CG, AH, and GC at the genus level. ^***^P < 0.001 and ^****^P < 0.0001.

**Figure 3:**
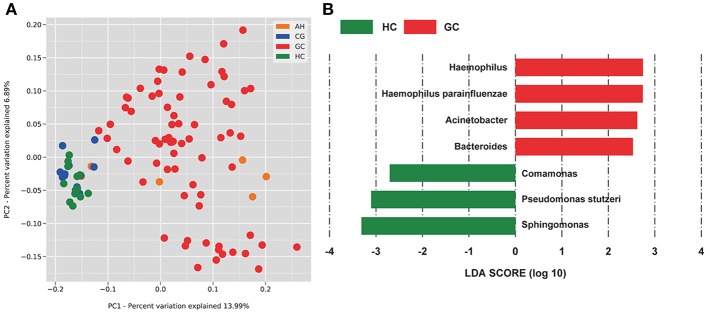
PCoA and LEfSe analysis of the microbiome between healthy control (HC) and patients with gastric cancer (GC). **(A)** Unweighted UniFrac PCoA. **(B)** Histogram of the LDA scores computed for different abundance levels between HC and GC. GC-enriched taxa are indicated with a positive score (red), whereas HC-enriched taxa show negative scores (green). Only taxa achieving an LDA significant threshold > 2 are shown.

The OTUs shared by at least half of the samples in each group are shown in the Venn diagrams that were constructed to evaluate the shared OTUs (Figure 2A). There were 886 shared OTUs in HC, 881 in CG, 597 in AH, and 466 in GC, and 397 OTUs were shared among all samples. Alpha diversity of the bacterial community was compared among CG, AH, GC, and HC. Figure 2B shows that the observed OTU number was significantly higher in HC (880.2 ± 48.76) and CG (882.5 ± 63.23) than in AH (546 ± 211.3) and GC (542.5 ± 96.59). Further analysis about the overall microbial compositions showed that Proteobacteria, Actinobacteria, and Firmicutes were the dominant phyla, representing over 95% of the total phyla in all groups (Figure 2C). Proteobacteria was the most abundant bacterial phylum in CG, AH, GC, and HC groups (57.45 ± 6.07, 62.92 ± 8.79, 58.61 ± 13.82, and 60.75 ± 1.69%), followed by Actinobacteria (25.27 ± 4.02, 24.9 ± 9.14, 24.4 ± 12.62, and 25.76 ± 1.84%) and Firmicutes (13.71 ± 10.02, 8.26 ± 2.47, 12.26 ± 8/33, and 10.03 ± 1.21%). At the genus level (Figure 2D), *Pseudomonas* was most abundant in AH (12.5 ± 9.33%), GC (11.7 ± 9.94%), and HC (10.82 ± 2.86%), whereas *Sphingomonas* was the most abundant in CG (12.06 ± 1.7%). We evaluated beta diversity among four groups using principal coordinate analysis (PCoA) based on the unweighted values (Figure 3A). Results demonstrated that PC1 explained 13.99% and PC2 6.89% of the total variation. To statistically support the visual clustering of the bacterial communities in PCoA analysis, different groups were examined using Adonis. Results indicated that the structure of the serum microbiome in GC was significantly different from those of other groups (Supplemental Table 2).

To identify specific bacterial taxa associated with GC, we compared serum microbiota using LEfSe analysis based on non-parametric factorial Kruskal-Wallis (KW) sum-rank test between GC and HC. Figure 3B shows that seven genera or species distinguished the serum microbial communities between two groups according to the criteria of LDA ≥ 2.5 and *P* < 0.05. *Haemophilus* (order Pasteurellales, family Pasteurellaceae), *Acinetobacter* (order Pseudomonadales, family Moraxellaceae), *Bacteroides* (order Bacteroidales, family Bacteroidaceae), and *Haemophilus parainfluenzae* were enriched in GC group. And in HC group, *Sphingomonas* (order Sphingomonadales, family Sphingomonadaceae), *Comamonas* (order Burkholderiales, family Comamonadaceae), and *Pseudomonas stutzeri* (order Pseudomonadales, family Pseudomonadaceae) were also enriched in the HC group, which indicated serum microbial dysbiosis in GC.

### The Clinical Indices With Serum Microbiome in GC

**Figure 4:**
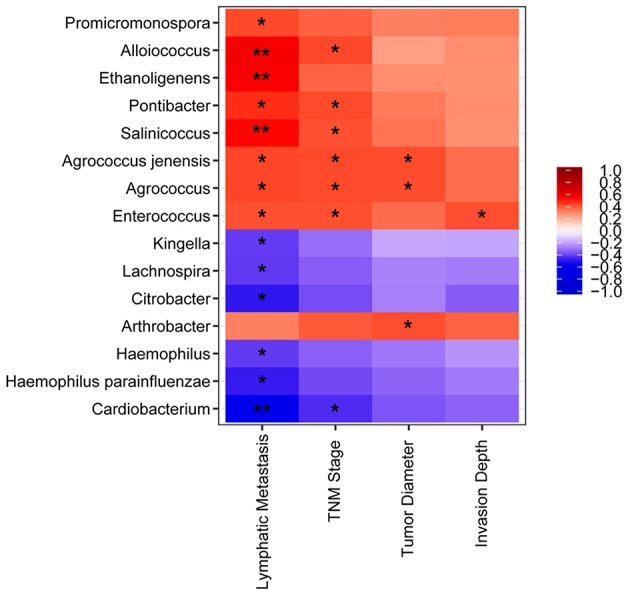
Heatmap of spearman correlation analysis among the serum microbiota of gastric cancer and clinical relative indices. ^*^P < 0.05, ^**^P < 0.01.

We detected a correlation between serum microbiome in GC (genus and species level) and clinical parameters used Spearman's rank correlation coefficient. Interestingly, 15 genera and species correlated significantly with clinical indices, including TNM stage, lymphatic metastasis, tumor diameter, and invasion depth (Figure 4). Lymphatic metastasis exhibited a significant positive correction with *Alloiococcus* (*r* = 0.532, *P* < 0.01), *Ethanoligenens* (*r* = 0.526, *P* < 0.01), and *Salinicoccus* (*r* = 0.498, *P* < 0.01), and negative correlation with *Cardiobacterium* (*r* = −0.533, *P* < 0.01). TNM stage showed positive correlation with *Agrococcus* (*r* = 0.431, *P* < 0.05), *Pontibacter* (*r* = 0.431, *P* < 0.05), *Alloiococcus* (*r* = 0.436, *P* < 0.05), *Enterococcus* (*r* = 0.423, *P* < 0.05), *Agrococcus jenensis* (*r* = 0.431, *P* < 0.05), and *Salinicoccus* (*r* = 0.419, *P* < 0.05). TNM stage correlated negatively with *Cardiobacterium* (*r* = 0.447, *P* < 0.05). Tumor diameter was positively correlated with *Agrococcus* (*r* = 0.428, *P* < 0.05), *Arthrobacter* (*r* = 0.424, *P* < 0.05), and *A. jenensis* (*r* = 0.428, *P* < 0.05). Invasion depth showed positive correlation with *Enterococcus* (*r* = 0.426, *P* < 0.05). Furthermore, serum microbiome did not show any correlation with gender, age, cancer location, differentiation, general classification (Bormann), distal metastasis, or fecal occult blood tests (all *P* > 0.05).

### Microbiome Present in the Serum of Patients With GC and HC

**Figure 5:**
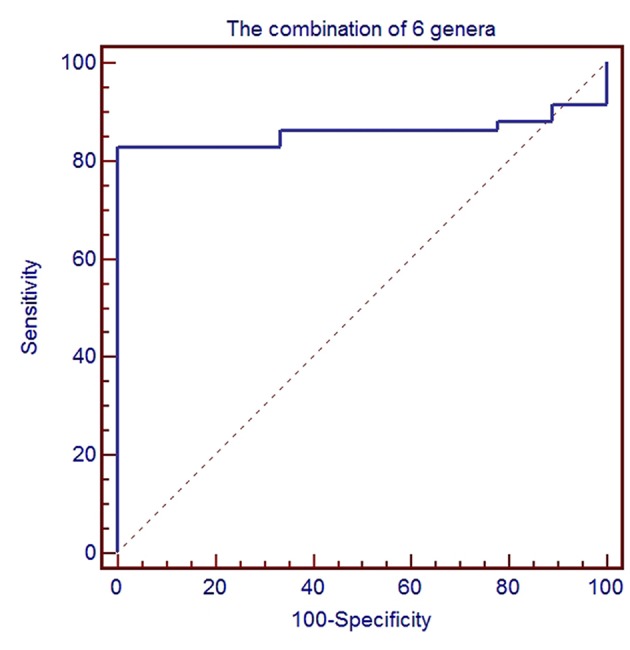
ROC analysis for the predictive value of gastric cancer based on six microbiota including *Acinetobacter, Bacteroides, Sphingomonas, Comamonas, H. parainfluenzae*, and *P. stutzeri* between healthy control and gastric cancer.

To further evaluate GC disease status, we constructed a classifier model that could specifically identify GC samples from HC samples. Based on LDA selection (Supplemental Table 3), a total of 6 genera or species were selected for ROC analysis, including *Acinetobacter, Bacteroides, Sphingomonas, Comamonas, H. parainfluenzae*, and *P. stutzeri*. First, we randomly selected 80% data as a training set using random-number generators in SPSS software. ROC was performed according to the abundance of the bacteria between GC and HC, and the combination of six microbial biomarkers yielded an AUC of 0.858 (95%CI: 0.751–0.931) with sensitivity 82.8% and specificity 88.9% (Figure 5). Then the remaining 20% was used as a validation set. The validation accuracy was 76.47%. The AUC of remaining 20% data was 0.814 (95%CI: 0.717–0.912). The sensitivity and specificity were 76.9 and 75.0%. These results showed that the combination of six microbial biomarkers achieved a high classification power for GC and HC.

### Serum Microbiome in Patients With Gastric Cancer Lymphatic Metastasis (GC-LM) or Non-lymphatic Metastasis (GC-NLM)

**Figure 6:**
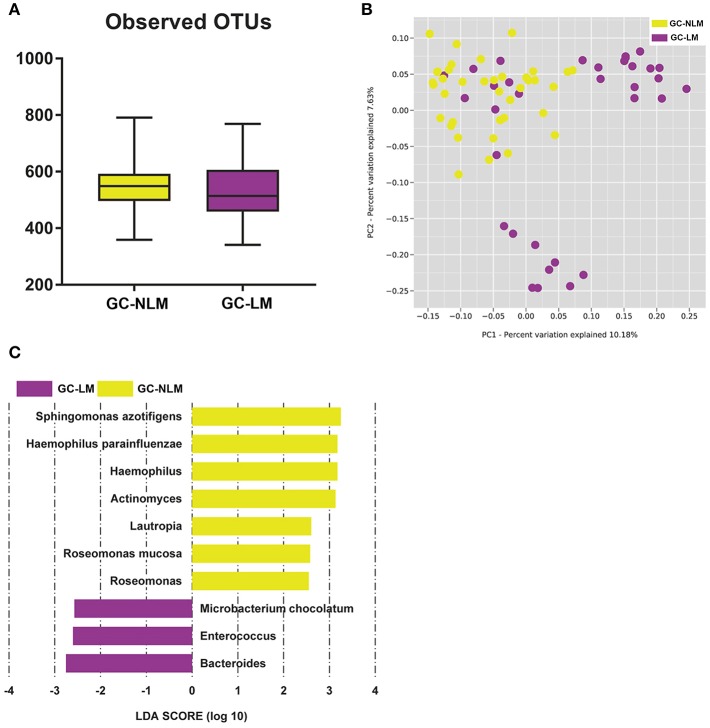
Characteristics of microbial community composition in patients with gastric cancer lymphatic metastasis (GC-LM) or non-lymphatic metastasis (GC-NLM). **(A)** Observed OTUs in GC-LM and GC-NLM. **(B)** Unweighted UniFrac PCoA. **(C)** Histogram of the LDA scores computed for different abundance levels between GC-LM and GC-NLM.

To identify the differences between microbiome in GC-NLM and GC-LM, we stratified samples for further analysis. Seventy one GC samples were stratified into GC-NLM (38 cases) and GC-LM (33 cases) according to metastasis or non-metastasis status. Comparison of the alpha diversity between two groups did not reveal significant differences in the number of observed OTUs between GC-NLM and GC-LM (Figure 6A, *P* = 0.29). Moreover, we used unweighted PCoA analysis to investigate beta diversity between GC-NLM and GC-LM, and Figure 6B indicated that the structure of serum microbiota in GC-NLM correlated weakly with that in GC-LM (Adonis, *R*^2^ = 0.0582, *P* = 0.0001). In addition, LEfSe analysis was used to further identify different compositions of microbiota and identify significant biomarkers in GC-NLM and GC-LM (Figure 6C). Three species (*Sphingomonas azotifigens, H. parainfluenzae, Roseomonas mucosa*) and 3 genera (*Haemophilus, Lautropia, Rosemonas*) were enriched in GC-NLM. *Microbacterium chocolatum* and 2 genera (*Enterococcus* and *Bacteroides*) were enriched in GC-LM.

## Discussion

To date (2019), cell culture or molecular tests are the main methods used for the identification of bacterial species (22). However, the culture method has some limitations for the slow-growing or fastidious bacteria, for example, identification was complicated or time consuming (23). Our laboratory (24) has optimized the current procedure for the identification of bacteria using matrix-assisted laser desorption/ionization-time of flight mass spectrometry (MALDI-TOF MS) and developed an easy, fast, and accurate combinatorial method. However, our procedure does not work well for a relatively low number of samples, especially for non-fermenters and enterococci. Currently, 16S rRNA high-throughput sequencing offers a powerful culture-independent approach for studying the underlying diversity of microbiata in tissues, especially in blood. Aberrations in blood microbiota have been identified in several diseases, including Parkinson's disease (13), renal disease (25), cardiovascular disease (15), and liver fibrosis (26). However, to our knowledge, data regarding the composition of blood or serum microbiome in GC is lacking so far. In the present study, we detected bacterial DNA in the serum of patients with GC. This is consistent with the observations of several previous studies that have also focused on the composition of serum/plasma microbiome in certain diseases, such as systemic inflammation (27), cirrhosis (17), and cardiovascular disease (16). Thus, we paid more attention to the difference in serum microbiome, which contributes to our understanding of the process of GC development.

Classical GC development or the “normal- chronic gastritis (CG)-atypical hyperplasia (AH)-gastric cancer (GC)” progressions, is a complex process. In our study, the bacterial communities in HC, CG, AH, and GC groups were structurally different, with alpha diversity (OTU numbers) decreasing from HC to GC. Our observations were supported by previous results, which showed that patients with GC had also lower bacterial diversity, and the main microbiota was characterized by Proteobacteria (69.3%), Firmicutes (14.7%), Bacteroidetes (9.0%), and Actinobacteria (4.3%) (9). The reason for lower diversity in GC may be that chronic *H. pylori* infection decreased acid secretion in gastric mucosa, providing a suitable microenvironment for the successful establishment of a new microbiota that contributes to malignant transformation. Reduced microbial diversity has now been considered as a feature of diseased states, such as inflammatory disease or cancer (28, 29). These results illustrated a global shift in microbiota from HC to GC, and the altered microbiota might play a vital role in the initiation and development of GC.

As serum microbiota has limited contact with the gastrointestinal tract, the source of these microbiota remains unclear. The tight barrier at the intestinal-vascular interface can prevent bacteria from entering into circulation from the gut under normal conditions (30). However, the integrity of the barrier can be compromised under certain pathological conditions, which results in microbial translocation into circulation. For example, Santiago et al. (17) characterized and compared the microbial composition of stool, serum, and ascitic fluid in patients with cirrhosis using 16S rRNA high-throughput sequencing. The results showed that the three sites shared few common microbial taxa. They suspected microbial translocation, which was confirmed by the presence of bacterial DNA in ascitic fluid and blood derived from the gastrointestinal tract. A similar instance of microbial translocation has also been investigated in HIV+ patients (31). Koren et al. (32) showed that oral bacteria might translocate into the blood of patients with atherosclerosis. Recently, the relationship between patients with GC and the oral microbiome has been reported (33, 34). However, the microbiomes in serum or blood have not been investigated so far. In terms of serum microbiome composition, we observed three dominant phyla, including Proteobacteria, Actinobacteria, and Firmicutes, which mostly represent the intestinal commensals. These results were similar to those of previous studies on gastric tissue or gastric fluid (9, 35, 36). The above studies provide an indication that the microbiome should be investigated not only in different subjects, but also in the oral cavity, serum, and gut of the same subjects with GC to understand the origin and role of the serum microbiome. As we know, the imbalance of the immune system state has been confirmed in some diseases, as well as gastric cancer. Immune cells can regulate the morphology and function of GC cells, which changing the integrity of gastric tissue. This enables microbiota to translocate into blood. Therefore, the change of serum microbiota in GC might be an indirect rather than a causal effect. After all, this is just a speculation, and further study about the underlying mechanism is warranted.

At the genus level, our results showed that *Pseudomonas* was the most abundant, which are in accordance with the results of other studies (11, 36). Importantly, using LEfSe analysis, we were able to identify specific bacterial taxa associated with GC, including the genera *Haemophilus, Acinetobacter, Bacteroides*, and *H. parainfluenzae*. Interestingly, the abundance of *Bacteroides* and *Acinetobacter* have been reported to increase, and are associated with gastric carcinogenesis (37, 38), as well as colorectal cancer (39). A Previous research revealed that *Haemophilus* is the major component of the human gastric microbiota (36), and *H. parainfluenzae* is detected in 63.3% (19/30) patients with lung cancer (40). This demonstrates the consistency of bacterial expression in serum and tissues, indicating an important role in the development of GC. Our results also demonstrated that the genus *Sphingomonas, Comamonas*, and *P. stutzeri* were enriched in HC. This is consistent with the results of Xuan et al. (41). However, another study showed that the abundance of *Sphingomonas* is increased in colitis associated cancer (42). We speculated that this might be related to differences in tumor types. Reports show that products isolated from *P. stutzeri*, such as pigment PY3 (43) and L-asparaginase (44), possess anticancer potential and induce cell apoptosis in cervical cancer (HeLa), liver cancer (HepG2), and leukemia (Jurkat). Therefore, we speculate that the enrichment of microbiota in HC, compared to GC, might be the result of confrontation with tumors during GC progression. In addition, some microorganisms are also enriched in GC tissue, including *Streptococcus, Prevotella, Lactobacillus*, and *Lachnospiraceae* (6, 9, 45). However, we did not detect these in the sera in our study. We speculated that this might be due to differences in sample type, primers, or the sequencing platform. In addition, differences in geographical environment or diet might also be responsible.

The integration of data from the most relevant genera that characterized GC and HC allowed us to investigate the correlation between serum microbiome in GC and clinical parameters. Previous studies in human or INS-GAS mice have shown that altered gastric microbiota (*H. pylori* or non*-H. pylori*) may participate in GC progression via various pathways, such as those involved in increasing the production of N-nitroso compounds (46, 47). We also identified 15 serum genera and species closely related to TNM stage, tumor diameter, invasion depth, and lymphatic metastasis. This indicated that these microorganisms, including *Enterococcus, Agrococcus*, might be involved in gastric carcinogenesis. Metastasis is not only an important feature of GC, but also one of the criteria in tumor staging (48). In particular, lymphatic metastasis is the most common form of metastasis in GC. Thus, we stratified samples for further analysis on metastasis, and two genera (*Enterococcus* and *Bacteroides*) were enriched. This showed excellent capacity to distinguish between GC-NLM and GC-LM. Interestingly, *Enterococcus* has been related to several cancers, such as colon cancer, and lung cancer (49, 50). Strickertsson et al. observed that *Enterococcus* infection down-regulated the expression of the miR-17-92 cluster in gastric adenocarcinoma cell (51). The colonization of the *Bacteroides* species ASF519 was linked to GC risk (37). These results implied that serum microbiota contributed to GC development.

Recent studies have reported that microbiota can be used to predict the status of GC. Ferreira et al. (9) calculated the microbial dysbiosis index (MDI) to differentiate between gastritis and gastric carcinoma with 10 taxa. A scoring system was also designed to screen suspected patients with GC via oral microbiome detection (34). The concept of microbiome acting as a predictive tool for specific diseases or cancer has been established by several compelling studies. Qin et al. demonstrated that microbiota might be useful for classifying type 2 diabetes (52). Some studies on cancer have also identified and validated microbiota for the diagnosis of diseases such as colorectal cancer (53), hepatocellular carcinoma (54), and pancreatic carcinoma (28). Thus, we propose that serum microbiota may be used as a potential tool for early diagnosis of GC. In this study, we selected six microbiota by setting LDA ≥ 2.5 and *P* < 0.05, namely *Acinetobacter, Bacteroides, Sphingomonas, Comamonas, H. parainfluenzae*, and *P. stutzeri*. Importantly, the combination of the six microbiota achieved high AUC, which enabled differentiation between GC and HC and allowed development of a better predictive model for GC. Thus, this might constitute an easy-to-use sampling approach for the diagnosis and characterization of GC. The predictive value of the blood microbiome in some diseases has been recently investigated. Fifteen blood genera, including *Limnobacter*, were selected as the optimal marker set for Parkinson's disease and were associated with its clinical characteristics (13). In addition, the serum microbiome was considered as a live indicator of cirrhosis progression (17). In the future, 16S rRNA gene sequencing might be necessary for the detailed analysis of the microbial taxon and function in the serum of GC patients, which will provide novel insights into early diagnosis and therapy for GC.

In summary, our study illustrated the characteristics of the serum microbiome composition in patients with GC. This suggested that the serum microbiota might be a novel predictive tool for GC. However, this study could not determine whether these microbial changes were a cause or consequence of GC progression, and further studies are required to elucidate the specific role of different microorganisms. Therefore, the transplantation of culturable bacteria in animal models is essential to validate the possible causative relationship between serum microbiota and GC progression in future.

## Ethics Statement

This study was carried out in accordance with the recommendations of the Ethics Committee of Qilu Hospital of Shandong University (Approval No: KYLL-2015-097) with written informed consent from all subjects. All subjects gave written informed consent in accordance with the Declaration of Helsinki. The protocol was approved by the Ethics Committee of Qilu Hospital of Shandong University.

## Author Contributions

LZ and YZ: concept and design of study. DW, YY, and KX: collect clinical samples. ZD, BC, XZ, JY, ML, RZ, and MZ: performed the experiment. ZD, BC, and HP: data analysis and interpretation. YZ: supervision. ZD and BC: manuscript writing. All authors revised and approved the final version of the manuscript.

### Conflict of Interest Statement

The authors declare that the research was conducted in the absence of any commercial or financial relationships that could be construed as a potential conflict of interest.
